# Cerebellar cognitive disorder parallels cerebellar motor symptoms in Friedreich ataxia

**DOI:** 10.1002/acn3.51079

**Published:** 2020-06-08

**Authors:** Gilles Naeije, Myriam Rai, Nick Allaerts, Martin Sjogard, Xavier De Tiège, Massimo Pandolfo

**Affiliations:** ^1^ Laboratoire de Cartographie Fonctionnelle du Cerveau ULB Neuroscience Institute Université libre de Bruxelles (ULB) Brussels Belgium; ^2^ Department of Neurology CUB Hôpital Erasme Université libre de Bruxelles (ULB) Brussels Belgium; ^3^ Laboratoire de Neurologie Expérimentale ULB Neuroscience Institute Université libre de Bruxelles (ULB) Brussels Belgium

## Abstract

Dentate nuclei (DN) are involved in cerebellar modulation of motor and cognitive functions, whose impairment causes ataxia and cerebellar cognitive affective syndrome (CCAS). Friedreich ataxia (FRDA) disease progression relates to degeneration of the dentate nucleus and dentato‐thalamic pathways, causing cerebellar ataxia. Volumetric MRI also shows mild loss in the cerebellar cortex, brainstem, and motor cortex. Cognitive deficits occur in FRDA, but their relationship with ataxia progression is not fully characterized. We found a significant positive correlation between severity of patients’ ataxia and more marked CCAS as assessed with the CCAS‐Scale. This relation could be related to progressive DN impairment.

## Introduction

Friedreich ataxia (FRDA) is the most common autosomal recessive ataxia in Caucasians.^1^ Most patients are homozygous for the hyperexpansion of an intronic GAA triplet repeat in the frataxin (FXN) gene,^2^ which repress FXN expression via an epigenetic mechanism.^3^ Neural systems show marked variability in their vulnerability to FXN deficiency and in their timing of involvement.^4^, ^5^ The proprioceptive system is affected first,^6^, ^7^ followed by progressive cerebellar and pyramidal pathology.^8^ At the cerebellar level, FRDA is mainly characterized by progressive loss of large neurons in the dentate nucleus (DN),^9^, ^10^ whose axons form the dentato‐thalamic pathway connecting the cerebellum with a wide array of neocortical areas. In addition to motor control, such cortico‐cerebellar loops play an important role in many perceptual and cognitive processes.^11^, ^12^ Yet, cognitive disorders in FRDA are often overlooked due to the fact that they are relatively subtle and do not cause obvious functional impairment.^13^ Also, the screening tools commonly used to detect cognitive abnormalities, such as the Mini Mental State Evaluation (MMSE) and the Montreal Cognitive Assessment (MOCA), are normal (MMSE)^14^, ^15^ or slightly abnormal (MOCA)^16^ and failing to capture the specific features of FRDA patients' cognitive impairment. However, when comprehensively evaluated, FRDA patients, though having no frank intellectual disability or dementia, present with reduced cognitive processing speed, lower performance in language and visuospatial tasks, impaired executive functioning and poorer ideas generation.^17^ This pattern of cognitive dysfunction observed in FRDA patients corresponds well to the cerebellar cognitive affective syndrome (CCAS),^18^ defined by altered executive function, visuospatial cognition, affect regulation, and language, over and above speech. Recently, a dedicated bedside CCAS‐Scale built with a combination of pencil and paper tests and taking less than 10 minutes to realize, has been developed and validated as CCAS diagnostic tool.^19^ In FRDA patients, the use of this scale could provide crucial information about their cognitive alterations related to cerebellar impairment as most validated clinical assessment tools used in FRDA,^20^, ^21^ focus on motor signs and symptoms.

Here, we postulate that cognitive impairment in FRDA corresponds to a progressive form of CCAS.^18^ To test that hypothesis, we sought an association between cerebellar ataxia motor symptoms, as assessed with the scale for the assessment and rating of ataxia (SARA) and the cognitive function of FRDA patients evaluated by the CCAS‐Scale^19^ that would support shared underlying mechanism.

## Subjects and methods

### Subjects

Nineteen FRDA patients from the Brussels site of the European Friedreich Ataxia Consortium for Translational Studies (EFACTS) clinical study^22^, ^23^ participated in the study. Of note, three patients were heterozygous for a GAA1 repeat expansion and a point mutation in the FXN gene (Table 1).

**Table 1:** Characteristics of the included FRDA patients.

Age (mean, [range], years)	30 [12‐54]
SARA (median, [range])	23 [7.5‐38]
Disease duration (median ± standard deviation; years)	15 ± 11
GAA1 (median, [range])	668 [445‐912]

SARA, score on the Scale for the Assessment and Rating of Ataxia; GAA1, number of GAA1 triplet expansion on the shortest allele.

### Clinical assessment

Cerebellar ataxia was assessed with the SARA, which includes eight items evaluating gait, stance, sitting, speech, finger chase test, nose‐to‐finger test, fast alternating movements of the hands, and heel‐to‐shin test.^20^ We used the CCAS‐Scale to assess cognitive function. The CCAS‐Scale is composed of 10 items: a semantic fluency task, a phonemic fluency task, a category switching task, a forward digit span, a backward digit span, a cube drawing task, a verbal registration task, a similarities task, a Go No‐Go task, and an affect evaluation.^19^ A raw score is obtained for each task, with a minimum passing score. The number of failed tests determines the likelihood that the subject has CCAS: three or more failed tasks make a definite CCAS, two a probable CCAS and one a possible CCAS. The raw score ranges from 82 (sum of minimum passing scores for each item on the scale) to 120 (sum of maximum scores for each item) is not diagnostic but provides quantitative values in each tasks that can be used for longitudinal follow‐up as patients can have definite CCAS (three failed test items) with a total raw score that falls in the 82–120 range. Subjects without CCAS are not supposed to fail any task.^19^ Patients were tested with both scales in the same session.

### Ethical statement

All participants were included in the study after written informed consent. The study had prior approval by the CUB Hôpital Erasme Ethics Committee and was performed in accordance with the Declaration of Helsinki.

### Statistical analysis

Spearman rank correlation tests were used to assess possible relations between CCAS‐Scale total score and the number of failed items, SARA score, the size of GAA1 triplet expansion, the age of symptoms onset, and disease duration. Patients with FXN point mutations were not included in GAA1 analyses. Results were considered statistically significant after correction for multiple correlation (n = 6) at *P* < 0.008.

## Results

All patients failed at least one CCAS‐Scale item: four patients failed one item, three patients failed two items and 12 patients failed three or more items. The number of failures, the mean ± standard deviation of the raw scores, the minimum passing score and the maximum raw score for each item are presented in Table 2.

**Table 2:** CCAS‐Scale detailed results.

	FRDA	Minimum Passing Score	Maximal Raw score
Semantic fluency (number of words)	17 ± 6	>15	26
Phonemic fluency (number of words)	10 ± 3	>9	19
Category switching (numbers of co)	10 ± 3	>9	15
Digit span Forward (correct numbers of a series)	6 ± 1	>5	8
Digit span Backward (correct numbers of a series)	4 ± 1	>3	6
Cube	12 ± 5	>11	15
Verbal registration	14 ± 2	>10	15
Similarities	8.5 ± 0.5	>6	8
Go‐No Go	1.7 ± 0.5	>0	2
Affect	4 ± 1.5	>4	6
Failed Items^1^	3 ± 1.6		0
Raw Score^2^	86 ± 13		120

^1^ ≥ 3 = definite CCAS, 2 = probable CCAS, 1 = possible CCAS.

^2^ Ranges from 82 (sum of minimum passing scores for each item on the scale) to 120 (sum of maximum scores for each item). Of notice, as items have different weights, definite CCAS (three failed test items) can occur with total raw score that falls in the 82–120 range.

**Figure 1:**
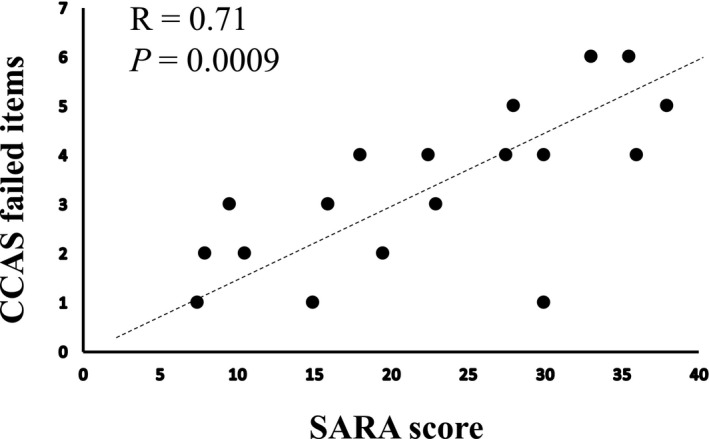
Scatterplot and linear regression line of SARA scores vs. CCAS‐Scale number of failed items.

Patients with higher CCAS‐Scale raw scores had lower SARA scores and vice versa (r = −0.63, *P* = 0.004). There was no significant correlation between the CCAS‐Scale total score and disease duration (r = −0.36, *P* = 0.14) or age of symptoms onset (r = 0.04, *P* = 0.87) or the size of GAA1 expansion (r = −0.29, *P* = 0.33). Patients who failed a higher number of items had higher SARA scores (r = 0.71, *P* = 0.0009, Figure 1) and longer disease duration (r = 0.49, *P* = 0.004). There was no significant correlation between the number of failed items and the size of GAA1 expansion (r = 0.51, *P* = 0.08) or the age of symptoms onset (r = 0.02, *P* = 0.93).

## Discussion

The main finding of this study is that all FRDA patients failed at least one item of the CCAS‐Scale, whose severity tightly correlates with the SARA score.

Despite the relatively small sample, this finding is likely to be generally valid as our population shares similar clinical characteristics with FRDA patients from large follow‐up cohorts^22^ and with smaller populations specifically investigated for cognitive disturbances.^16^, ^24^, ^25^

There is a scarce but growing number of studies investigating cognitive functioning and cerebellar symptoms in FRDA and in other diseases with cerebellar impairment. Studies using comprehensive neuropsychological testing showed that FRDA patients display a wide range of cognitive abnormalities, affecting conceptual thinking, verbal abilities, response time on Simon Task,^26^ selective attention and inhibition,^15^, ^27^ and emotion recognition.^28^ These cognitive impairments have been associated with altered resting state functional connectivity,^16^, ^24^ and, in a study^26^, with posterior cerebellar lobe atrophy, while correlation with clinical and genetic parameters was less consistently observed. It is possible that the complexity of datasets generated by extensive and lengthy neuropsychological test batteries (over 90 minutes in many of the studies) made correlations arduous and translation in clinical practice difficult^14^, ^15^. Thus a simple and rapid test to assess cognitive function in FRDA is desirable. As shown here, the CCAS‐scale, a pencil, and paper test that can be completed in less than 10 minutes, allows a quick and sensitive detection of cognitive alterations in FRDA patients, with a quantitative assessment of its severity.

This study demonstrates that this cognitive evaluation, specifically designed for patients with cerebellar diseases, is well suited to assess cognitive impairment in FRDA compared to commonly used cognitive screening tests like the MoCA, which fails to discriminate between FRDA patients and healthy subjects.^29^

The positive correlation between the number of failed CCAS‐Scale items, disease duration and ataxia severity as assessed with the SARA, revealed a close link between motor and cognitive disturbances in FRDA. The characteristics of the CCAS‐scale, which defines CCAS in terms of failed items regardless of total raw score, explain why no significant correlation was found between raw scores and disease duration, even though raw scores provide a quantitative assessment of the patients’ performance that can be used for eventual follow‐up.^19^ We cannot exclude, however, that the lack of correlation between CCAS‐Scale raw scores and disease duration may also be a false negative reflecting a statistical type II error due to our limited sample size.

The pathophysiology of cognitive impairments revealed by the CCAS‐scale in FRDA may relate to progressively altered cerebello‐cortical connections because of DN impairment and consequently of the dentato‐thalamic pathway. The functional strength of these connections is illustrated by the phenomenon of crossed cerebellar diaschisis, which refers to the hypometabolism and reduced activity affecting a cerebellar hemisphere as a result of a contralateral supratentorial lesion, or a cortical region as a result of a cerebellar lesions or disconnection.^30^ Positron emission tomography (PET), functional magnetic resonance imaging (fMRI), and magnetoencephalography (MEG) data also indicated widespread changes in brain networks in FRDA,^29^, ^31^ with impaired cerebello‐cortical functional connectivity.^16^, ^17^, ^32^ These findings provide a potential pathophysiological substrate for the occurrence of cognitive disturbances in FRDA. Interestingly, in our FRDA patients, the severity of cognitive impairment is closely paralleled by the severity of ataxia. This contrasts with the dichotomy between cerebellar motor and non‐motor symptoms reported in other cerebellar pathologies where functional and lesional studies described a segregation between the cerebellar anterior lobe, responsible for sensory‐motor functions, and the cerebellar posterior lobe responsible for cognitive processes.^33^ In FRDA, cerebellar cortical atrophy is mild, occurs late in the evolution of the disease, and mainly affects the posterior lobe, particularly Lobule IX. By contrast, DN impairment is severe, progressive, and occurs more early in the evolution of the disease. Based on these considerations, DN impairment probably explains the parallel worsening of cognitive and motor deficits in FRDA.

Finally, as practical implication, our findings indicate that the CCAS‐Scale is a sensitive tool to quantify cognitive disturbances in FRDA and a potential outcome measure in clinical trials.

## Conflict of Interest

No author discloses conflicts of interest.
